# Chinese herbal injections for unstable angina pectoris: A protocol for systematic review and network meta-analyses

**DOI:** 10.1097/MD.0000000000010142

**Published:** 2018-03-23

**Authors:** Fengwen Yang, Jiahan Zou, Xuemei Li, Long Ge, Jinhui Tian, Myeong Soo Lee, Junhua Zhang

**Affiliations:** a Evidence-Based Medicine Center, Tianjin University of Traditional Chinese Medicine; b Baokang Hospital of Tianjin University of Traditional Chinese Medicine, Tianjin; c Evidence-Based Medicine Center, Lanzhou University, Lanzhou, China; d Clinical Research Division, Korea Institute of Oriental Medicine, Daejeon, Republic of Korea.

**Keywords:** Chinese herbal injection, network meta-analysis, unstable angina pectoris

## Abstract

**Background:**

Chinese herbal injections (CHIs) have been commonly used in the treatment of unstable angina pectoris (UAP) in China. However, there is no consensus or evidence on how to select CHIs for patients with UAP. The choice often depends on the personal experience or preference of clinician. This study aims to compare the effect of different CHIs for UAP using Bayesian network meta-analysis (NMA).

**Methods:**

A systematic search will be conducted in PubMed, the Cochrane Central Register of Controlled Trials, Embase, Chinese Biomedical Literature Database, China National Knowledge Infrastructure, and Wanfang Data from inception to February 2018. We will include randomized controlled trials (RCT) regarding CHIs in the treatment of UAP. Quality of included RCTs will be assessed according to the Cochrane Handbook 5.1.0. A Bayesian NMA will be performed with WinBUGS 14 to compare the efficacy of different CHIs. GRADE will be used to assess the quality of evidence.

**Results:**

The results of this NMA will be submitted to a peer-reviewed journal for publication.

**Conclusion:**

Our study will generate evidence for CHIs in the treatment of UAP and help clinicians make more accurate therapeutic schedule. In addition, it might provide suggestions for Chinese medicine clinical practice or guideline.

## 1 Introduction

Unstable angina pectoris (UAP) is a kind of acute coronary syndrome, with the characteristics of severe chest pain and long duration, it is likely to aggravate to myocardial infarction and lead to an increase in mortality.^[1]^ So, treatment should begin as soon as possible once symptoms or forebodings of UAP are onset. Anti-ischemic, antiplatelet, and antithrombotic therapies are usually recommended as the conventional pharmacotherapy in guideline.^[2]^ However, drawbacks like adverse effects and drug resistance often occur with the current medications.

Traditional Chinese Medicine (TCM), which has a history of more than 2000 years, has got widespread clinical applications in east and south Asia. Research indicates that TCM might be used in the prevention of cardiovascular disease as a complementary and alternative intervention.^[3]^ Chinese herbal injections (CHIs), which have the characteristic of rapid absorption and high bioavailability,^[4]^ play an important role in the medical system in China, especially in emergency treatment.

Several systematic reviews and meta-analyses have been conducted to assess the effect of single CHI for UAP.^[5–8]^ However, it is difficult to tell the difference among the CHIs by those pairwise meta-analyses since they were all designed in comparison with conventional western medicine. Network meta-analysis (NMA) is popularly used to evaluate healthcare interventions. It has the advantage of allowing indirect comparisons of multiple interventions for estimation and ranking their orderings even though direct head-to-head comparison studies are lacking.^[9]^

This study is a protocol of comprehensive NMA on different kinds of CHIs for the treatment of UAP.

## 2 Methods

### 2.1 Study registration

The study protocol has been registered on PROSPERO CRD 42018088370.

### 2.2 Eligibility criteria

#### 2.2.1 Type of study

Randomized controlled trials (RCTs) regarding CHIs for the treatment of UAP will be included for analysis. There will be no limitations on language, year of publication, or duration of study follow-up.

#### 2.2.2 Participants

Adults (aged 18 years or older) with UAP, which should be confirmed according to the diagnostic criteria,^[10–12]^ patients with acute myocardial infarction or after percutaneous coronary intervention will be excluded. No limitations exist in gender, race or nationality.

#### 2.2.3 Interventions

Interventions involving any CHI for the treatment of UAP are eligible. Conventional pharmacotherapy can be used together with CHIs. Conventional pharmacotherapy includes anti-ischemic, antiplatelet, antithrombotic therapies, and so on. The following 3 forms are eligible: CHI a versus CHI b; CHI + conventional pharmacotherapy versus conventional pharmacotherapy; CHI a + conventional pharmacotherapy versus CHI b + conventional pharmacotherapy. Conventional pharmacotherapy in 2 groups of 1 RCT should be same.

#### 2.2.4 Outcomes

The primary outcomes include mortality and acute cardiovascular events. The secondary outcomes include frequency of angina pectoris attacks, hypersensitive C-reactive protein and adverse drug events. RCTs reporting on at least one primary outcome will be included.

### 2.3 Data source

A comprehensive search will be performed in both English and Chinese database involving PubMed, the Cochrane Central Register of Controlled Trials, EMBASE, Chinese Biomedical Literature Database, China National Knowledge Infrastructure, and Wanfang Database from inceptions to February 2018. Language restriction not exists in this study. Search terms are: unstable angina pectoris, Chinese herbal injection and others. Full details of the Search strategy in PubMed are:

#1 (“Angina, Unstable”[MeSH Terms]) OR (“Unstable Angina∗”[Title/Abstract]) OR (“Unstable Angina Pectori∗”[Title/Abstract]) OR (“Angina at Rest”[Title/Abstract]) OR (“Preinfarction Angina∗”[Title/Abstract]) OR (“Myocardial Preinfarction Syndrome∗”[Title/Abstract])

#2 (“Medicine, Chinese Traditional”[MeSH Terms]) OR (“Traditional Chinese Medicine”[Title/Abstract]) OR (“Chinese Traditional Medicine”[Title/Abstract]) OR (zhongyi[Title/Abstract]) OR (zhongyao[Title/Abstract])

#3 (Injections[MeSH Terms]) OR (Injection[Title/Abstract]) OR (Injectables[Title/Abstract]) OR (Injectable[Title/Abstract]) OR (Injections[Title/Abstract])

#4 #2 AND #3 AND #1

### 2.4 Study selection

ENDNOTE X7 literature management software will be used to manage search records. First, the titles and abstracts of each record retrieved will be screened in ENDNOTE by 2 independent authors (FY and JZ) to identify relevant studies according to eligibility criteria. Then, full texts of all potentially relevant studies will be obtained and reviewed for further assessment. Disagreements will be resolved by discussion or consultation of a third author (JZ). When multiple articles are from the same data set, we will select the paper with the most complete data. In addition, the references of included studies will be tracked to identify other relevant studies. The reasons for exclusion of trials in full-text review will be reported in the excluded studies list. A data spreadsheet will be developed with Microsoft Excel 2007 to collect relevant information. The information, including eligible studies characteristics (e.g., first author, year of publication, country where the study was conducted), participant characteristics (e.g., gender, age, sample), interventions (e.g., name of CHI, duration, frequency of drugs) and outcomes will be extracted and entered into the spreadsheet.

### 2.5 Risk of bias assessment

The risk of bias of included studies will be evaluated by 2 independent reviewers (LG and JT) according to the Risk of Bias Tool (ROB) in Cochrane Handbook 5.1.0.^[13]^ If consensus cannot be reached, the third author (JZ) will be consulted. Bias in RCTs will be evaluated for 7 domains: method of random sequence generation (selection bias), allocation concealment (selection bias), participant and personnel blinding (performance bias), outcome assessment blinding (detection bias), incomplete data (detection bias), selective reporting (detection bias), and other bias. Each RCT will be classified as having a high, low, or unclear risk of bias.

### 2.6 Statistical analysis

#### 2.6.1 Pairwise meta-analyses

We will perform pairwise meta-analyses using Stata 13 software. Odds radio (OR) with 95% confidence interval (95% CI) will be carried out for dichotomous data while mean difference (MD) with 95% CI for continuous data. Potential heterogeneity across the included studies will be assessed by χ^2^ test and *I*^2^ test. If *I*^2^ ≥ 50% and *P* ≤ 0.1, it suggests that there is statistical heterogeneity. Then, subgroup analysis or meta-regression will be conducted to explore possible sources of heterogeneity if the number of included RCTs is sufficient. Sensitivity analysis of the included studies will be employed if the sample size of RCT is less than 50 or the baseline between groups is different. If *I*^2^ < 50% and *P* > 0.1, which infers that there is no statistical heterogeneity, and the Mantel–Haenszel fixed model will employed for meta-analysis. In addition, Begg's and Egger's funnel plot method will be performed to help distinguish asymmetry due to publication bias when applicable.^[14,15]^ If the data are not appropriate for quantitative analysis, we will describe and summarize the evidence instead.

#### 2.6.2 Network meta-analyses

The NMA will be performed in a Bayesian hierarchical framework using Markov Chain Monte Carlo method in WinBUGS 14 (MRC Biostatistics Unit, Cambridge University, UK).^[16]^ Three Markov chains will run simultaneously with different arbitrarily chosen initial values. We will first generate 50,000 simulations for each chain, and these samples will be discarded as “burn-in.” Then posterior summaries will be based on 100,000 subsequent simulations. We will also use node splitting method to explore the inconsistency between direct and indirect comparisons if a loop connecting 3 or more arms exist.^[17,18]^ Surface under the cumulative ranking (SUCRA) will be used to summarize the probability values, SUCRA has possible values ranging from 0% to 100%, and the higher value indicates the better efficacy.^[19]^ Comparison-adjusted funnel plots will also be conducted using Stata 13 software to explore the effects of the sample size among intervention networks. ^[20,21]^

### 2.7 Quality of evidence

The Grading of Recommendations Assessment, Development and Evaluation (GRADE) will be used to assess the quality of evidence for the main outcomes,^[22]^ it is classified into 4 levels: high level, moderate level, low level, and very low level. Flaws in risk of bias, indirectness, imprecision, inconsistency, and publication bias will be sought. This step will be conducted through the online guideline development tool (GDT, http:// gdt. guidelinedevelopment.org/).

## 3 Discussion

Although a few studies have assessed the effective of the individual CHI, the differences among these CHIs are still lack of evaluation and comparison. To the best of our knowledge, no network meta-analyses comparing the effect of CHIs for the treatment of UAP have been conducted recently. This NMA is expected to provide a ranking of the CHIs for UAP. We hope that the results of this NMA will help clinicians make more accurate treatment decisions and promote further research into TCM treatments for UAP. We also wait that the result would concern the interest of the government or policymakers, since this might help them make a better choice of the treatment that should be covered by insurance. This protocol is designed in accordance with guidelines for network meta-analysis protocols^[23]^ and the procedures of this NMA will be performed in adherence to the PRISMA extension statement for reporting of systematic reviews incorporating network meta-analyses of healthcare interventions.^[24]^ The results of this NMA will be submitted to a peer-reviewed journal for publication.
